# Environmental Polymorphism Registry: Banking DNA to Discover the Source of Susceptibility

**DOI:** 10.1289/ehp.114-a408

**Published:** 2006-07

**Authors:** Angela Spivey

Walking outside on a day when ozone levels are at “code orange” doesn’t bother one person, but for someone else, it can result in chest pain, coughing, wheezing, or lung and nasal congestion. Why? Polymorphisms, tiny interindividual variations in genes, may be part of the reason. Providing a pool of information to help researchers determine how these variations interact with the environment to cause disease is the ultimate goal of the Environmental Polymorphism Registry (EPR), which is sponsored by the NIEHS and conducted in collaboration with the University of North Carolina at Chapel Hill’s General Clinical Research Center.

Any North Carolinian over 18 years of age can donate a sample—about a tablespoon of blood—to the EPR. Rather than recruiting donors with a particular disease, the EPR aims to gather, over five years, samples from 20,000 people who represent the general state population. The regional nature of the effort facilitates recruitment and follow-up.

“Recruitment is monitored to ensure that the EPR population is representative of the North Carolina population,” says Patricia Chulada, one of the four principal investigators of the EPR and a health scientist administrator at the NIEHS. “If we see deficiencies in certain groups, then we can increase efforts targeted to those particular groups.”

This approach will help researchers find out which polymorphisms are most common. “We want to look at people’s genetic material and find variations, and then go back and figure out what those variations mean,” says another EPR principal investigator, Paul B. Watkins, a professor of medicine at UNC–Chapel Hill and director of the General Clinical Research Center.

Chulada and Perry Blackshear, the NIEHS director of clinical research, initiated the registry by approaching Watkins and Susan Pusek, director of training and career development at the General Clinical Research Center. Watkins says the institute—and Blackshear himself—realized that “this is an essential direction of research to understand why some people are healthy and some are sick.”

There are multiple DNA registry efforts in the United States. Two major DNA banking efforts include Northwestern University’s NUgene Project and the Marshfield Clinic’s Personalized Medicine Research Project, both launched in 2002. International DNA banks are even more common, Chulada says. For instance, Iceland’s deCODE Project has recruited more than 80,000 subjects and has published findings on genes associated with arthritis and many other common conditions.

The EPR is unique, however, because it is designed to focus on environmentally responsive genes—those that increase the risk of disease when combined with an environmental exposure. The registry was created with the express intent of facilitating clinical studies of polymorphisms in these genes. Being affiliated with the NIEHS, where scientists are already studying such interactions, makes the EPR a natural resource for these investigations.

## Protecting Participants

Unlike with anonymous DNA databases, EPR donors provide their names and contact information so they can be asked to participate in follow-up studies if their DNA contains a polymorphism of interest. Participation in follow-up studies is optional, and donors can drop out of the database at any time.

Donors learn about the steps taken to ensure confidentiality in a 6-page consent form. Study interviewers at recruitment tables also discuss this information with potential donors, Chulada says.

Donors’ names and other information are stored separately from samples. When a sample is collected, it’s assigned a personal identification number. The code key that links the sample to identifying information is kept separate from the sample and from all other data in a computer system that’s password-protected. Access to this system is limited to only a few people directly involved in the EPR. Researchers can obtain contact information for potential participants only after approval by the EPR Oversight Committee.

To receive samples, researchers must sign a material transfer agreement, in which the researcher’s institution agrees to several conditions. “They can only use the samples for what they outlined in the agreement,” Chulada says. “They can’t give the samples to others. And they have to destroy the samples within a certain amount of time [which varies on a case-by-case basis].”

In addition, the NIH has granted the EPR a Certificate of Confidentiality, which protects researchers from being required, even by subpoena, to disclose research data or other information about an individual to an outside party such as an insurance company, an employer, or a civil or criminal court. “This is another layer of protection built into this system,” Chulada says.

## Stepping Up Recruitment

The EPR has already accumulated about 4,000 samples—not far behind the 5,000 collected by NUgene since its launch. The EPR’s goal of 20,000 samples is the minimum needed to conduct certain types of studies with adequate statistical power, Chulada says. For example, if a researcher was interested in a rare genetic variant that occurs in only 1% of the population, the variant should be present in 200 samples from a registry of 20,000. “That would give us adequate statistical power to test for a phenotypic association of low to moderate effect, depending on other factors,” Chulada says.

When the EPR began, it recruited exclusively at two clinics at UNC–Chapel Hill. It has since expanded recruitment to Rex Hospital in Raleigh and is applying for approval to recruit at Duke University Medical Center in Durham. However, Chulada says, “Although recruiting at medical clinics gave us a diverse population in terms of health and other characteristics, we learned that we could increase both recruitment rates and diversity by recruiting outside of the clinic setting.”

A recruitment fair held for five days at the NIEHS campus in Research Triangle Park yielded about 420 donors. “We were ecstatic with the response of the NIEHS community,” Chulada says. The general public also can donate through study drives at corporations and health fairs in Research Triangle Park. Potential participants can visit the EPR web-site (http://dir.niehs.nih.gov/direpr/) to find out about upcoming drives.

## A DNA Goldmine

John Hollingsworth, a scientist working in the Environmental Lung Disease Group in the NIEHS Laboratory of Respiratory Biology, is one of the first investigators to apply for use of EPR samples. Hollingsworth and colleagues want to identify people who have a polymorphism in a certain gene, Toll-like receptor 4 (*TLR4*), known to be important in innate immune responses.

*TLR4* was first identified as a candidate gene for response to ozone by NIEHS scientist Steven Kleeberger, leader of the Environmental Genetics Group in the Laboratory of Respiratory Biology. Subsequently, Hollingsworth and colleagues have demonstrated that mice deficient in *TLR4* are protected against airway hyper-responsiveness after exposure to ozone. “We want to determine if this gene is important in people in the biologic response to inhaled ozone,” Hollingsworth says. “We’re trying to validate what we’ve seen in mice in a human cohort.”

Hollingsworth calls the EPR a “gold-mine.” He says, “It’s a perfect situation. We have a cohort willing to be genotyped, rather than doing a mass screening of people for a single project, which is what we’ve had to do in the past.”

## Figures and Tables

**Figure:**
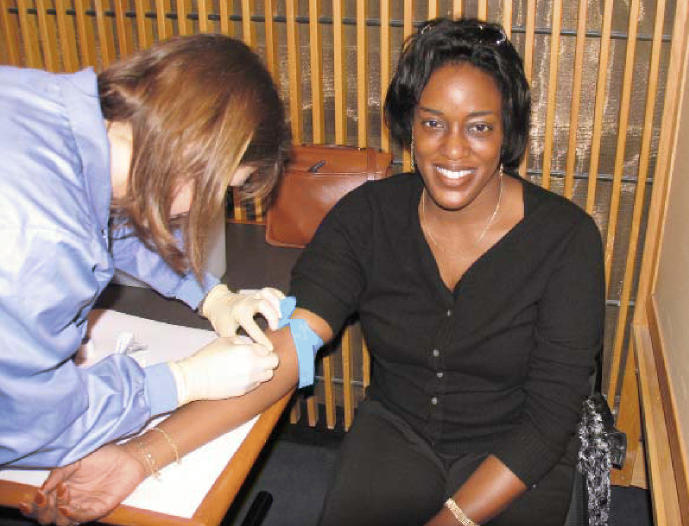
A little prick for a big cause The Environmental Polymorphism Registry aims to collect 20,000 blood samples that will be studied to help determine how genetic differences may result in disease.

